# FEM Analysis of Buckled Dielectric Thin-Film Packaging Based on 3D Direct Numerical Simulation

**DOI:** 10.3390/mi14071312

**Published:** 2023-06-26

**Authors:** Seonho Seok

**Affiliations:** Center for Nanoscience and Nanotechnology (C2N), University-Paris-Saclay, 91400 Palaiseau, France; seonho.seok@c2n.upsaclay.fr

**Keywords:** direct simulation, buckling, FEM, packaging, thin-film

## Abstract

This paper presents a 3D direct numerical simulation of buckled thin-film packaging based on transferred elastic thin-film wrinkling bonded on a compliant polymer ring. The mode change of the fabricated thin-film cap is found by measuring the thin-film cap shape at different times after Si substrate debonding. The conventional linear and nonlinear buckling simulations are not adequate to understand the behavior of the thin-film buckling mechanism creating such packaging cap mode change. Direct buckling simulation is recently reported as an easy and useful numerical wrinkling simulation method. A novel 3D FEM model of a thin-film package suitable for direct 3D buckling simulation is built to reduce the mode mixture between different buckling modes. Buckling modes of the packaging cap are investigated in terms of elastic moduli of package materials and applied strain due to thermal expansion coefficient difference. Based on the simulation results, it is found that there are two main modes in the fabricated thin-film buckling package determining the shape of the transferred thin-film packaging cover depending on the elasticity ratio between the cap and sealing ring materials. The mode shift from wrinkling cap mode to out-of-plane cap mode due to applied strain along a polymeric sealing ring is found.

## 1. Introduction

Microelectromechanical system (MEMS) devices need specific packages to house and protect the packaged device during wafer handling, wafer dicing, or plastic molding [1]. Transfer packaging techniques attract more and more interest because it delivers packaging caps from a carrier wafer to a device wafer. As packaging caps are separately prepared from the front-end process, the transfer packaging technique introduces minimal process compatibility issues compared to conventional packaging technologies [2,3]. Various transfer packaging technologies have been reported based on interface energy modification methods utilizing plasma-deposited fluorocarbon film [4], self-aligned monolayer (SAM) [5,6], solder transfer layer [7], etc. Various packaging cap materials such as metal, polymer, and Si materials can be transferred to the target device wafer by metallic or polymeric bonding techniques and debonding of the temporary carrier wafer. Because there are lots of materials associated with electronic packaging, FEM (Finite Element Method) simulations have been frequently used to find mechanical stress, strain, and reliability parameters of the electronic packages [8,9,10]. For example, FEM simulations of MEMS packaging and BCB (Benzocyclobutene) bonding have been presented with experimental verifications [11,12]. It is known that the die stress developed during the attachment can make the silicon substrate warp, and hence it seems to influence the performance of MEMS devices. Concerning the zero-level packaging, the capping near the device may generate significant stresses depending on the sealing and cap materials. Similarly, the BCB thin-film cap zero-level packaging can also have unwanted effects on the MEMS devices due to stress development resulting in package cap deformation and device chip deformation [13]. The principal source of this stress is the BCB cap’s residual stress developed at the bonding step, mainly due to the thermal expansion coefficient difference between BCB and the device materials. Similar to the complex packaging behavior mainly due to thermal stress, the transfer packaging process based on post-it-like debonding can also be simulated to understand polymer cap deformation and stress development during the debonding process [14]. The debonding of the carrier wafer has been implemented by defining the interface with the CZM (Cohesive Zone Model) method [15,16,17]. Through this modeling, we can find the cap deformation and the stress distribution of the BCB packaging cap transferred to the target wafer and, thus, the debonding process can be designed within the material’s mechanical reliability limit, such as tensile strength, elongation limit, etc.

In addition, the nonconventional material behavior, such as thin-film wrinkling on a polymer substrate, can also be modeled and simulated with FEM buckling modeling [18,19]. Thin-film wrinkling initiated by a mechanical mismatch with a supporting soft substrate can also be used to extract the material properties of the thin film [20,21,22]. As for finite element modeling associated with buckling instabilities, two steps of pre-buckling and post-buckling analysis with commercial software packages such as ABAQUS and ANSYS have been frequently used in the studies of the surface wrinkling phenomenon. Furthermore, modeling and simulation of thin-film packaging structures with the conventional approach are not easy due to the emergence of complex and various buckling modes due to the mixture of different basic buckling modes. As a solution for this troublesome aspect of the buckling FEM simulation, geometrical imperfection methods have been frequently employed by using mesh, geometry, and boundary condition perturbation techniques for studying surface instability. However, the existing imperfection approaches are relatively complex, and the implementation can be laborious due to free parameter calibration. Furthermore, the interpretation and verification of results are not straightforward, which makes it less practical for common users. To overcome the drawbacks of the geometrical imperfection methods, a simulation method that is direct, robust, and relatively easy to perform has been proposed where the pre/post-buckling simulations can be performed successfully in only one analysis step [23,24,25]. In addition, it can be implemented with any common finite element code and analysis platform. A direct and easy numerical simulation has been reported to overcome the drawbacks of the geometrical imperfection methods and, thus, it is applied to extract material properties extraction based on commercial scotch-tape surface wrinkling [26]. The reported literature has presented modeling methods of the basic structure and an elastic thin film on a polymeric substrate under uniaxial and biaxial loading conditions, and the resultant wrinkling patterns have been successfully created complying with theoretical patterns. Up to now, there is no report on buckling modeling and simulation in practical applications such as thin buckled thin-film packages based on an elastic thin film on polymeric sealing rings. To apply the direct numerical modeling method, the overlap part of the sealing has been intentionally removed to reduce the mode mixture between basic buckling modes. The established direct FEM model has successfully provided the wrinkling wavelength as a function of the material properties associated with surface wrinkling.

In this paper, a 3D direct numerical simulation of dielectric thin-film packaging is proposed to understand the buckling behavior of the dielectric thin-film packaging cap transferred on a polymeric sealing ring. The transfer packaging process is briefly introduced in Section 2. The theoretical background of thin-film wrinkling on a compliant substrate is explained in Section 3, and the FEM model for direct numerical simulation is described in Section 4. Finally, the conclusion will be made in Section 5.

## 2. Thin-Film Transfer Packaging Process

Transfer packaging technology attracts more and more interest because it could minimize the process compatibility of packaging with the device packaged by a thin film or polymer cap encapsulation. Until now, many different cap materials such as metal, silicon, and polymer have been transferred by various transfer methods based on hydrophobic coating, solder, and SAM (Self-Aligned Monolayer) coating. The most critical technological barrier of the transfer technique is to achieve clean debonding of the carrier substrate after the bonding process. To this end, a thin dielectric film has been transferred by using a SAM coating in order to achieve post-it-like debonding for wafer-level packaging technology.

**Figure 1:**
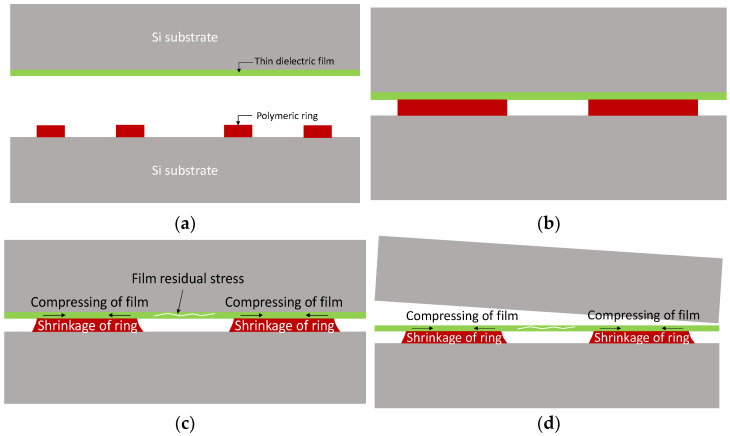
Thin-film transfer packaging. (**a**) Sample preparation before bonding; (**b**) Start bonding at 250 °C; (**c**) Bonding at 250 °C after 1 h; (**d**) Debonding of Si substrate.

Thin-film transfer packaging is depicted in Figure 1: (a) Thin elastic film is deposited on a Si substrate with hydrophobic surface modification. The BCB ring has been patterned on a Si substrate as a sealing material, which has only been soft-baked at 90 °C. (b) The two Si substrates have started the bonding at 250 °C with a Karlsuss bonding machine. Temperature and pressure profiles applied for the bonding are found in Ref. [27]. (c) During BCB curing at 250 °C for 1 h, the BCB ring is fully cured and, thus, its volume shrinkage becomes substantial. A mechanical mismatch between the thin direct film and the BCB ring creates a wrinkling pattern on the sealing ring part. (d) After the bonding process is completed, the Si substrate with a thin dielectric film is mechanically debonded. Because there is SAM (Self-Aligned Monolayer) for Si surface modification, the debonding of the Si substrate is quickly accomplished due to the cracking of the interface between the Si substrate and the dielectric film.

**Figure 2:**
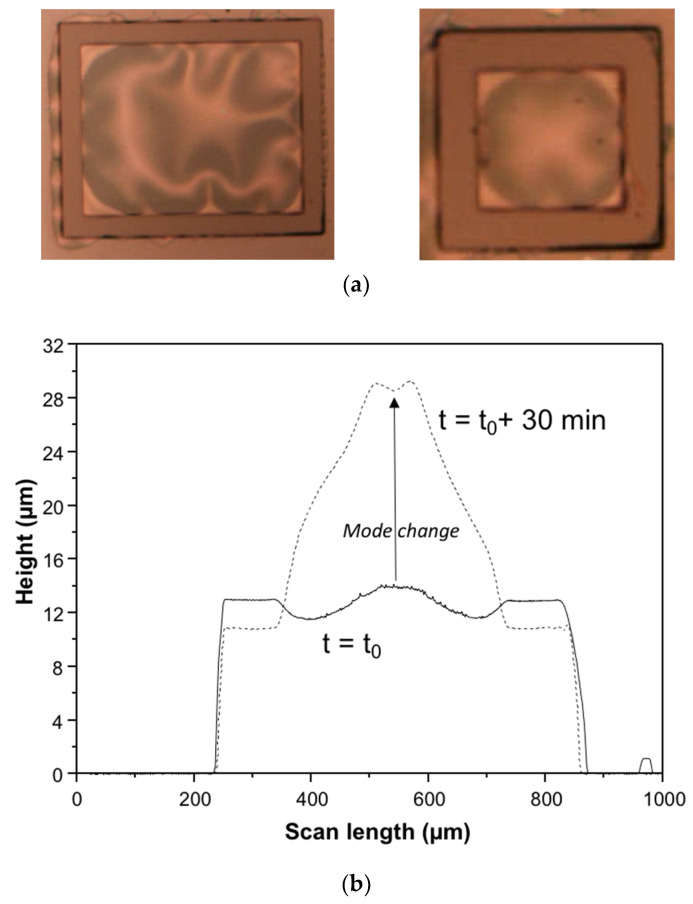
Transfer packaging results (modified from Ref. [27]). (**a**) Buckled thin film bonded on BCB ring (modified from Ref. [27]). (Transferred film thickness = 0.97 μm, BCB ring width = 150 μm, cavity size of small package = 400 μm × 400 μm); (**b**) Thin-film shape change as a function of time after debonding of carrier Si substrate.

Figure 2a shows the transferred buckling thin film on the BCB ring, and the surface wrinkling of the sealing ring was confirmed by the overhang of the thin film in the figure on the left. The thin-film cap shape change was found by measuring the thin-film cap profiles at different time intervals after Si substrate debonding, as shown in Figure 2b. It was also found that wrinkling of the thin dielectric film from the dielectric overhang occurred due to a mechanical mismatch between the dielectric film and the BCB ring. Interestingly, thin-film buckling occurred even if a flat Si substrate had been pressed against the sealing ring. As this wrinkling pattern is the initiating force for the buckling thin-film packaging, it was used for the modeling of the fabricated buckling thin-film package.

## 3. Theory of Thin-Film Buckling on Polymer Substrate

The buckled thin-film packaging is driven by wrinkling of the thin dielectric film bonded to the BCB ring, and thus, it was modeled and simulated with the direct 3D FEM method instead of 2D modeling. First, the theoretical background of elastic thin-film wrinkling will be briefly explained in the following.

The force balance approach for surface buckling instability of a thin film on a compliant substrate is briefly introduced here [28]. Considered a semi-infinite substrate under plane strain deformation, the classical equation for bending an elastic film on a compliant elastic substrate is given by Equation (1):

(1) $$\bar{E_{f}}I\frac{d^{4}z}{dx^{4}}+F\frac{d^{2}z}{dx^{2}}+kz=0,$$

where $\bar{E}$ = *E*/(1 − *ν*^2^) is the plane-strain modulus, *E* is the Young’s modulus, *v* is the Poisson’s ratio, *I* = *wh*^3^/12 is the moment of inertia (where *w* is the width of the film and h is its thickness), *F* is the uniaxially applied force or load, and *k* is the Winkler’s modulus of an elastic half-space (*k* = $\bar{E_{s}}w\pi /\lambda )$. The subscripts, *f* and *s*, denote the film and substrate, respectively.

As the buckling instability of interest here is the first sinusoidal mode, the film deflection can be described by Equation (2).

(2) $$z\left(x\right)=A\sin\frac{2\pi x}{\lambda }$$

Substituting Equation (2) into Equation (1) and solving for the applied force in the thin film on compliant substrate gives Equation (3).

(3) $$F=4\bar{E_{f}}I{\left(\frac{\pi }{\lambda }\right)}^{2}+\frac{\bar{E_{s}}w}{4}{\left(\frac{\pi }{\lambda }\right)}^{-1}$$

The film buckling wavelength can be found by minimizing *F* with respect to *λ* (or ∂F/∂λ=0):

(4) $$\lambda_{inf}=2\pi h{\left(\frac{\bar{E_{f}}}{3\bar{E_{s}}}\right)}^{\frac{1}{3}}$$

It should be noted here that the wavelength is only a function of the thickness of the film and the elastic properties of the film and substrate. Thus, the wrinkling wavelength can be used to determine the material properties of the thin film if the substrate material properties are known.

**Figure 3:**
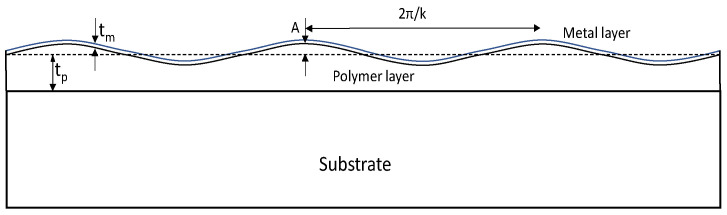
Geometry of thin buckled film on polymer layer.

Under finite polymer substrate thickness in a thin film on an elastic substrate, the buckling wavelength derived in Equation (4) should be modified considering the limited substrate thickness [29,30]. For a buckled bilayer of a thin film shown in Figure 3, the total free energy is given by the sum of the bending energy of the metal layer and the deformation energy of the underlying polymer layer. For a sinusoidal deformation in one direction, the low amplitude (*t_m_k* << 1) wave profile *w*(*x*) can be written as *w*(*x*) = A sin(*kx*), where tm is the metal thickness, *A* is the amplitude of the buckling wave, the buckling wavelength *λ* is 2π/*k*, and *k* is the wave number. Referring to the geometry of the thin film on a polymer layer given below, the free energy per unit area to bend a metal thin film having thickness *t_m_*, elastic modulus *E_m_*, and Poisson’s ratio *ν_m_* is given by

$$F_{bending}=\frac{E_{m}t_{m}^{3}}{48\left(1-\nu_{m}^{2}\right)}A^{2}k^{4}$$

For the free energy of deformation of an isotropic elastic film, the free energy expression approximated by the sum of the energy in the long wavelength limit (*t_p_k* << 1) and that in the short-wavelength limit (*t_p_k* >> 1) is

$$F_{Deformation}=\frac{E_{p}A^{2}}{4k^{2}t_{p}^{3}}+\frac{1}{6}E_{p}A^{2}k$$

where the subscript *p* indicates the polymer layer. For the small amplitude (*A* << *λ*) under consideration, the external strain *U* can be approximated by (*Ak*/2)^2^. Then total energy *F_t_* can be written as

$$F_{t}=\left(\frac{E_{m}k^{2}t_{m}^{3}}{12\left(1-\nu_{m}^{2}\right)}+\frac{E_{p}}{k^{4}t_{p}^{3}}+\frac{2E_{p}}{3k}\right)U$$

The intrinsic buckling wavelength can be obtained by minimizing the free energy with respect to the wave number and in a dimensionless form:

L=Y1+1+12YH313

where $L=\frac{\lambda_{fi}}{2\pi t_{m}}$, $Y=\frac{1}{2\left(1-\nu_{m}^{2}\right)}\times \frac{E_{m}}{E_{p}}$, and $H=\frac{t_{m}}{t_{p}}$.

## 4. Direct 3D FEM Modeling and Simulation

### 4.1. Uniaxial Loading of Thin Film/Polymeric Ring Stack

**Figure 4:**
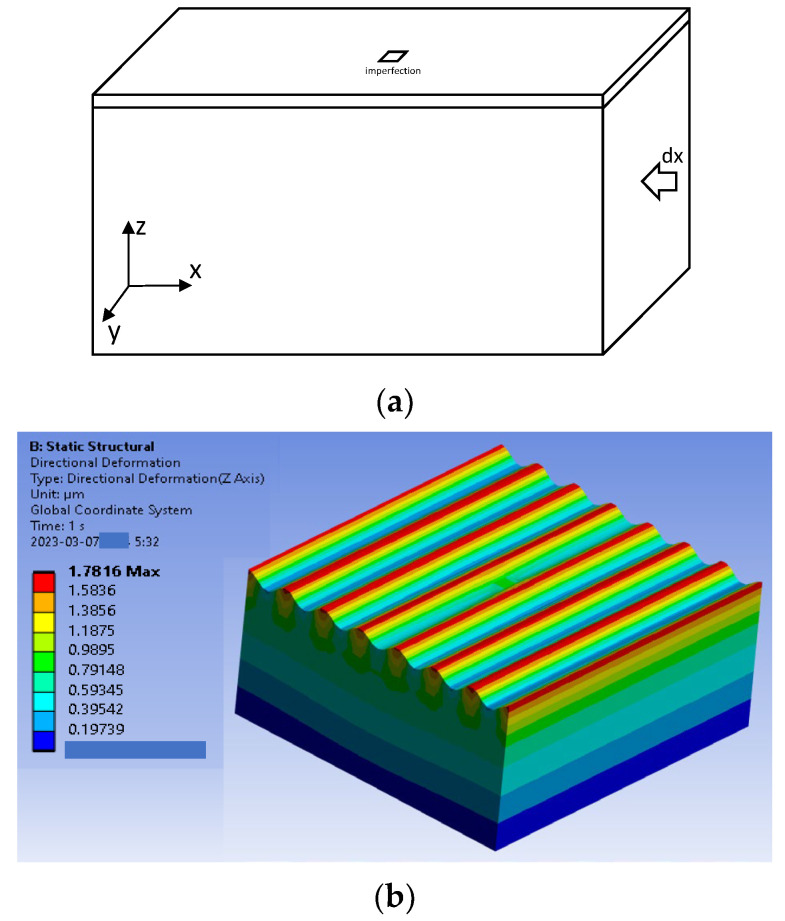
Model for 3D direct simulation. (**a**) 3D model; (**b**) Wrinkling pattern.

As previously shown in experimental results, the BCB sealing ring had created one-dimensional linear buckling due to its aspect ratio. Therefore, it was first checked with linear buckling modeling and simulation. The model and its boundary conditions of the dielectric film and BCB sealing ring stack are shown in Figure 4. As direct 3D modeling uses imperfections directly defined in the elements of the model, such as the previous work [31], the sealing ring was modeled and simulated to find the effect of imperfections on the wrinkling pattern. The model for the 3D direct simulation was composed of two layers, a thin dielectric film, and a BCB sealing ring, as shown in Figure 4a.

**Figure 5:**
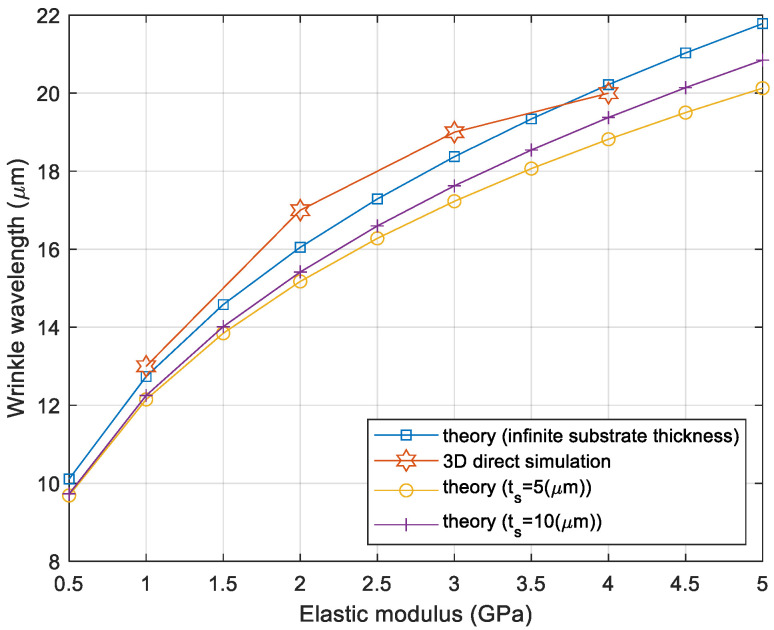
Wrinkling wavelength comparison: theory vs. 3D direct simulation.

**Figure 6:**
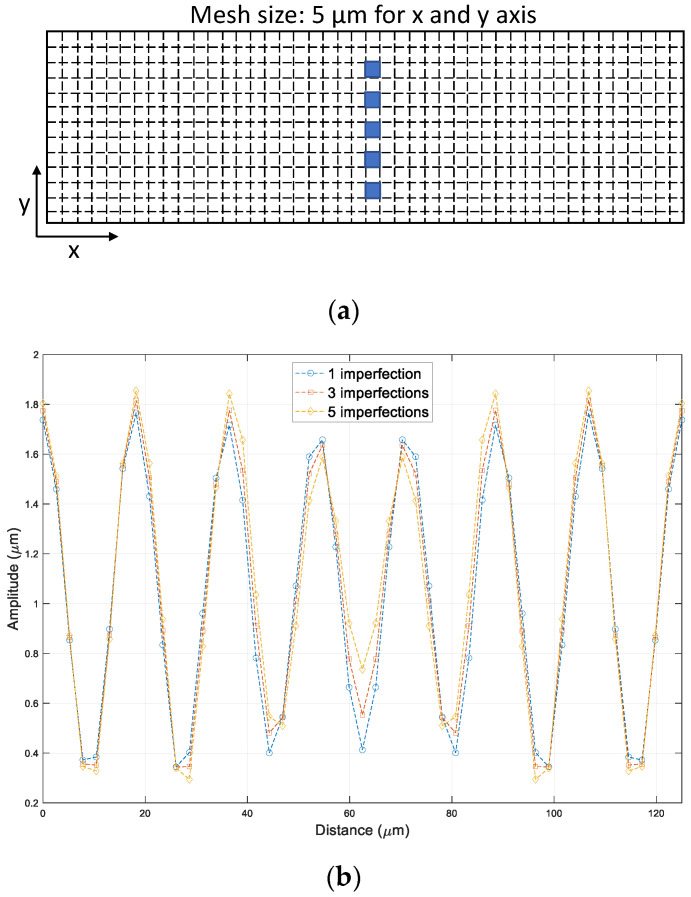
Wrinkling patterns vs. imperfection number. (**a**) Imperfection defined on top of polymer sealing ring; (**b**) Wrinkling patterns.

The model had a length of Lx = Ly = 125 µm, a thin-film thickness of tf = 0.5 µm, a polymer thickness of tp = 50 um, and the boundary conditions were Ux = 0 at x = 0, Uy = Uz = 0 at z = 0. The imperfection was defined at the center of the elements on top of the BCB sealing ring. Note that the mesh size was kept as a constant equal to λcr/3, where λcr is the critical wavelength when Es = 1GPa. Material properties of the model were Ef = 5 MPa, νf = 0.3, Es = 2 GPa, and νs = 0.4. As expected, the sinusoidal wrinkling pattern occurred by uniaxially applied displacement load, as shown in Figure 4b. It should be noted that the thin substrate effect was included in the comparison. The wavelength of the wrinkling pattern was studied as a function of the elasticity of the polymeric material substrate. The wavelength obtained from the 3D direct simulation had good agreement with the analytical calculation, as shown in Figure 5. For example, the elasticity of the thin film, 1 GPa, created a wavelength of 13 µm at given conditions. Afterward, the imperfection defined in a polymer substrate was essential for the 3D direction modeling and simulation, and thus, the dependence of the number of imperfections on the wrinkling pattern was investigated. As the sealing ring had a rectangular shape, the imperfections were defined along the width at its center line, as shown in Figure 6a. The number of imperfections 1, 3, and 5 were equally spaced imperfections that were manually selected and given with metallic material properties. The wrinkling patterns had sinusoidal waveforms for all the imperfection cases, and the wavelengths of the wrinkling patterns had no dependency on the number of imperfections, as presented in Figure 6b.

### 4.2. Buckled Thin-Film Package Transferred on Polymer Sealing Ring

**Figure 7:**
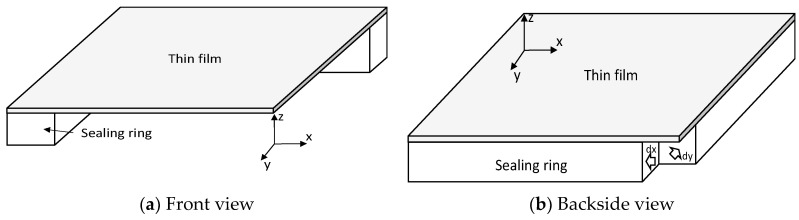
3D quarter model of buckled thin-film package. (**a**) Front view with symmetric surface; (**b**) Backside view with modified sealing ring to reduce buckling mode coupling.

As explained in the packaging process, the thin film transferred on the polymer sealing ring has shown two different profiles at certain time intervals. Evidently, there are two sources of the forces to determine the buckled thin film: (i) polymer sealing ring shrinkage resulting in wrinkling thin film and (ii) residual stress of the transferred thin film. Hence, it is necessary to study the effect of the two elements on the thin-film profile using FEM modeling. The buckled thin-film package has been modeled and simulated with a quarter model to reduce simulation time. Figure 7 shows the quarter models with the applied boundary conditions as follows. The symmetry planes create boundary conditions of Ux = 0 at x = 0 and Uy = 0 at y = 0, and the bottom surface has been constrained Uz = 0 to avoid rigid body motion. As explained in the previous section, the key parameter for the thin-film bucking is displacement (or strain) load which should be applied in the sealing ring of the package model. Therefore, the model has been slightly modified to avoid complex buckling modes by decoupling the buckling mode at the corner of the sealing ring, as shown in Figure 7b. The dimension and material properties of the thin film and sealing ring for the modeling are summarized in Table 1.

**Figure 8:**
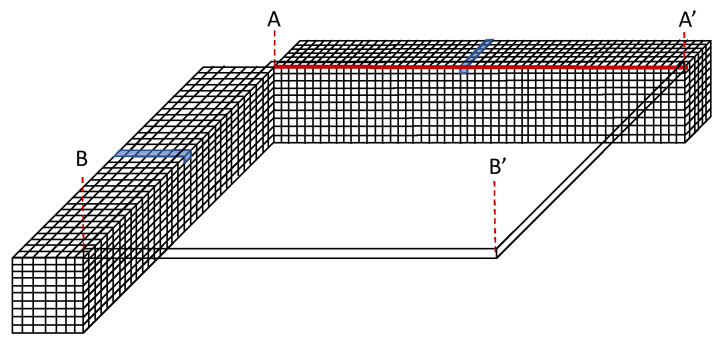
Imperfections defined in the sealing ring.

In a similar way to the previous section, imperfections in the sealing ring have been defined to perform the 3D direct buckling simulation. In this case, all the meshed elements of the top center of the sealing ring have been defined with imperfections, as shown in Figure 8. Given the imperfection, the applied displacements indicated in Figure 7b create the wrinkling of the thin nitride film due to a mechanical mismatch between the sealing ring and the thin film. Such wrinkling pattern on the sealing ring is one of the driving forces of the buckled thin-film packaging. The residual stress of the thin film should be another important factor for the final shape of the buckled thin-film package. The applied displacement was −1 µm in the x and y directions.

#### 4.2.1. Elasticity Ratio Ef/Es

**Figure 9:**
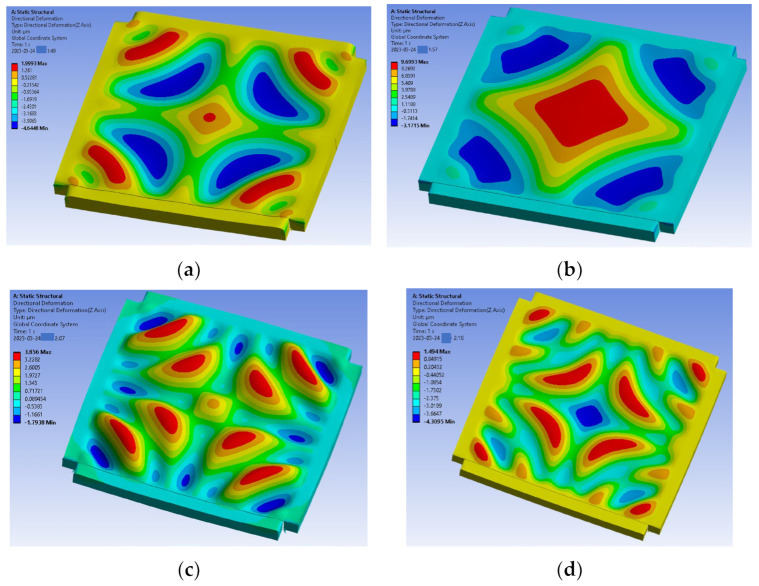
Thin-film wrinkling pattern as a function of the sealing ring’s material properties. (**a**) Es = 5 MPa; (**b**) Es = 10 MPa; (**c**) Es = 20 MPa; (**d**) Es = 40 MPa.

**Figure 10:**
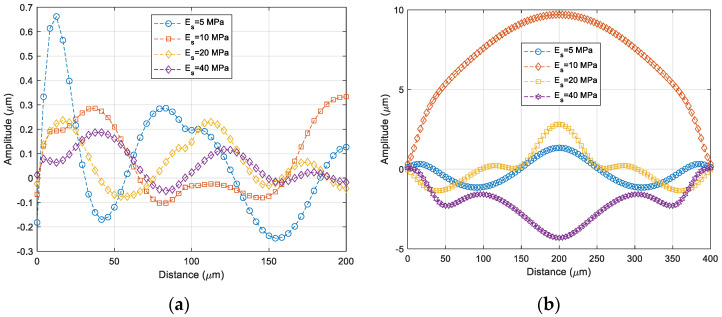
Wrinkling patterns as a function of elastic modulus of the polymer sealing ring. (**a**) AA′ of Figure 8; (**b**) BB′ of Figure 8.

Buckling patterns of the thin film depending on the ratio of Ef/Es have been investigated by varying the elasticity of the sealing ring. Figure 8 shows the buckled thin film depending on the elasticity of the sealing ring. It varies from 5 MPa to 40 MPa when the Young’s modulus of the thin film is 2 GPa. The wrinkling frequency of the thin film is inversely proportional to the Ef/Es ratio, as is the theoretical calculation. The increase in the wrinkling frequency has also been validated with the wrinkling patterns obtained at AA′ in Figure 8, as shown in Figure 9. This wrinkling pattern has been found just after Si carrier wafer debonding, as previously presented in Figure 2b. The measured wrinkling pattern is quite close to that of the simulation with Es is 5 MPa which is also validated from the cap profile at the center of the thin-film package shown in Figure 10b.

#### 4.2.2. Applied Strain

**Figure 11:**
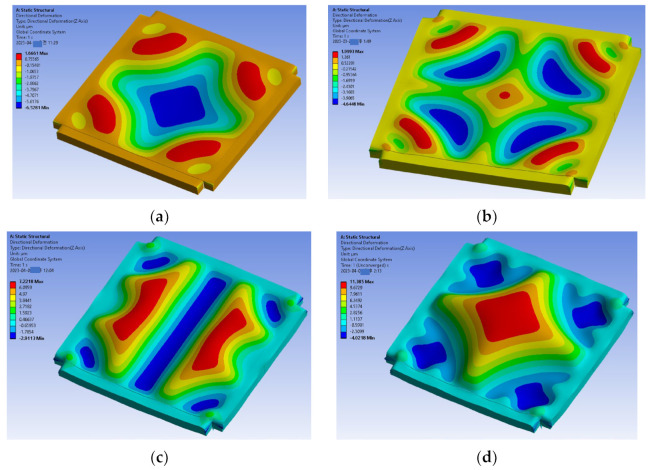
Thin-film wrinkling pattern as a function of applied strain. (**a**) εx = εy = 0.0025; (**b**) εx = εy = 0.005; (**c**) εx = εy = 0.0075; (**d**) εx = εy = 0.01.

**Figure 12:**
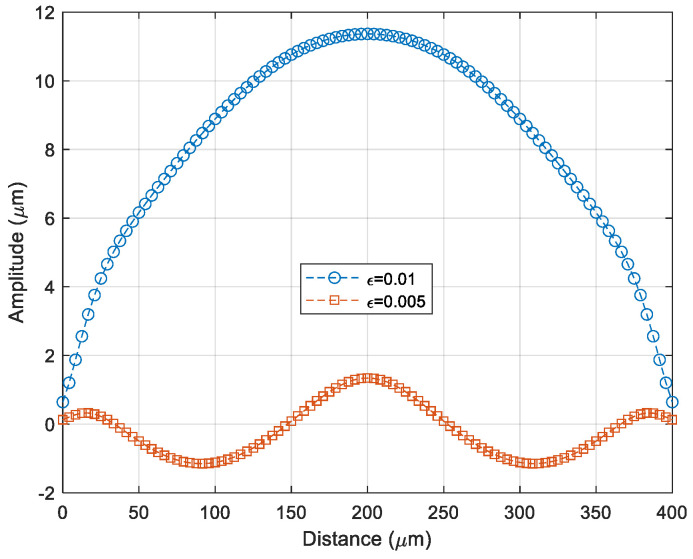
Packaging cap profile shift from the case of Figure 11b to the case of Figure 11d.

The strain developed in the polymeric sealing ring varies as a function of time after the debonding of the Si substrate. The Si substrate for the transfer of the thin film could prevent the ring from shrinking during the bonding process. The polymeric sealing ring should be fully cured during the bonding process. It is known that the volume shrinkage of the BCB polymer sealing ring is substantial after full curing. Therefore, the effect of the applied strain of the polymeric sealing ring on the thin-film buckling modes was studied. For this purpose, the case of Es with 5 MPa in the previous section was chosen because it has a similar cap shape to the fabrication result. Based on the simulation results, it is found that the thin-film packaging cap shape has been changed due to the applied strain, which is caused by the BCB sealing ring shrinkage. Experimental results have good agreement in the packaging cap shape shift from Figure 11b–d. The profiles of the wrinkling packaging cap of the two cases are shown in Figure 12.

## 5. Conclusions and Perspectives

A 3D direct modeling and simulation have been implemented to comprehend the behavior of the Si3N4 buckled thin-film package fabricated with a transfer technique. The mechanism of the buckled thin-film package is the mechanical mismatch between the elastic thin film and the polymeric sealing ring. Such a buckling phenomenon is simulated with commercial FEM software based on eigenvalue buckling and non-linear buckling sequences. The buckling simulation method provided by the commercial FEM software may not be suitable for understanding the behavior of the thin-film cap buckling due to the complex mode mixture among basic buckling modes. In this work, a fabricated thin-film package was modeled and simulated with the existing 3D direct buckling method. To achieve an efficient model, the 3D direct FEM model for the thin-film package was built to reduce such mode mixture of the buckling thin film and, thus, the buckling thin-film cap shapes were successfully extracted depending on mechanical parameters such as mechanical elasticities of the package materials and applied strain. Through the simulation, it was found that the experimental buckling cap shape of the thin-film package was mainly changed due to applied strain caused by the shrinkage of the polymer sealing ring. In conclusion, the direct geometrical imperfection method is an easy and versatile technique to make a buckling simulation of nonconventional mechanical structures, which can also be applied to other devices based on the buckling phenomenon.

## Figures and Tables

**Table 1:** Quarter model dimension and material properties.

Part Name	Dimension	Material Properties
Thin film	200 µm (L) × 200 µm (W) × 1 µm (H)	Ef = 2 GPa, νf = 0.3
Sealing ring	200 µm (L) × 25 µm (W) × 25 µm (H)	Es = 5 MPa, νs = 0.4

## Abbreviations

BCB	Benzocyclobutene
FEM	Finite Element Method
CZM	Cohesive Zone Model
SAM	Self-Aligned Monolayer
MEMS	Microelectromechanical system
